# The Art of Exploring Diatom Biosilica Biomaterials: From Biofabrication Perspective

**DOI:** 10.1002/advs.202304695

**Published:** 2023-12-03

**Authors:** Xiaojie Sun, Mengxue Zhang, Jinfeng Liu, Guangyan Hui, Xiguang Chen, Chao Feng

**Affiliations:** ^1^ College of Marine Life Science Ocean University of China 5# Yushan Road Qingdao Shandong Province 266003 China; ^2^ Department of Stomatology Qingdao Women and Children’s Hospital, Qingdao Qingdao 266034 China; ^3^ Department of Stomatology Qingdao Special Servicemen Recuperation Center of PLA Navy No.18 Yueyang Road Qingdao Shandong Province 266071 China; ^4^ Sanya Oceanographic Institute, Ocean University of China Yazhou Bay Science & Technology City Floor 7, Building 1, Yonyou Industrial Park Sanya Hainan Province 572024 P. R. China; ^5^ Laoshan Laboratory 1# Wenhai Road Qingdao Shandong Province 266000 China

**Keywords:** biofabrication, biomaterials, diatom biosilica, micro‐nanostructure

## Abstract

Diatom is a common single‐cell microalgae with large species and huge biomass. Diatom biosilica (DB), the shell of diatom, is a natural inorganic material with a micro‐nanoporous structure. Its unique hierarchical porous structure gives it great application potential in drug delivery, hemostat materials, and biosensors, etc. However, the structural diversity of DB determines its different biological functions. Screening hundreds of thousands of diatom species for structural features of DB that meet application requirements is like looking for a needle in a seaway. And the chemical modification methods lack effective means to control the micro‐nanoporous structure of DB. The formation of DB is a typical biomineralization process, and its structural characteristics are affected by external environmental conditions, genes, and other factors. This allows to manipulate the micro‐nanostructure of DB through biological regulation method, thereby transforming the screening mode of the structure function of DB from a needle in a seaway to biofabrication mode. This review focuses on the formation, biological modification, functional activity of DB structure, and its application in biomaterials field, providing regulatory strategies and research idea of DB from the perspective of biofabrication. It will also maximize the possibility of using DB as biological materials.

## Graphical abstract

Diatom biosilica (DB), the shell of diatom, is a natural inorganic material with a micro‐nanoporous structure. This review focuses on the formation, biological modification, functional activity of DB structure and its application in biomaterials field, providing regulatory strategies and research idea of DB from the perspective of biofabrication. It will also maximize possibility of using DB as biological materials.

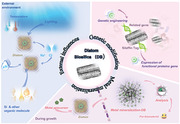

## 1 Introduction

Diatom, belonging to the plant kingdom, Bacillariophyta, is a kind of single‐celled eukaryotic algae with a chromatophore. It is often connected by several individual cells into various groups, which are distributed in fresh water and seawater. Marine diatoms alone have more than 150 000 species,^[^ ^1^, ^2^ ^]^ which can be divided into *Centric* and *Pennate* species.^[^ ^3^ ^]^ The *Centric* diatoms are generally round, radially symmetric, and mainly inhabit the upper and middle waters; however, the *Pennate* diatoms are long or navicular, symmetrical on both sides, and have benthic species. Diatom is an important primary producer in the ocean and the whole ecosystem, providing 20% of the global oxygen, accounting for 40% of the annual carbon production of marine organisms, which is about 20% of the global total annual carbon sequestration.^[^ ^4^ ^]^

Diatom cell walls is composed of organic material and biosilica (DB, the diatom shell, is composed of amorphous silica dioxide). DB's shape and pattern have intra‐specific conservation and inter‐specific differences. Its pore structure has high symmetry and regular size distribution. And it is composed of the upper and lower valves called the epitheca and hypotheca, respectively, and the girdle bands (gbs) of the side wall.

DB is formed through a biofabrication process, which is one of the basic biological processes for organisms to produce multifunctional and specific minerals.^[^ ^5^ ^]^ The general biofabrication process involves the synthesis of silica (biological silicification), the synthesis of calcium‐based biominerals (biological calcification), and the synthesis of iron‐based biominerals (biological magnetization). Many species that can synthesize rigid mineralized structures have been found in taxonomy. The biofabrication process occurs in single cells, advanced plants, animals, and humans, mainly during the formation of bones, teeth, shells or exoskeletons.^[^ ^4^ ^]^ And diatoms create silica cell walls with complex patterns through biofabrication. The process of diatoms converting soluble silicate into amorphous silica to form DB with complex structures is called silicification, that is biofabrication of diatoms. Besides, DB is a naturally produced micro‐nanosilica material whose formation process avoids the disadvantages of high energy consumption and high pollution in the chemical synthesis process. In terms of structure, DB also has unique porous 3D micro‐nanopatterns and high surface area, and their complex structure cannot be replicated in artificial synthesis at present.^[^ ^6^ ^]^ During the evolution of diatoms, DB structure has undergone adaptive changes. With unique optical and mechanical properties, DB plays an important role in diatom photosynthesis and predator resistance. The layered porous structure of DB also plays a role in separating, filtering, and eliminating nutrients and harmful micro‐organisms.

**Scheme 1:**
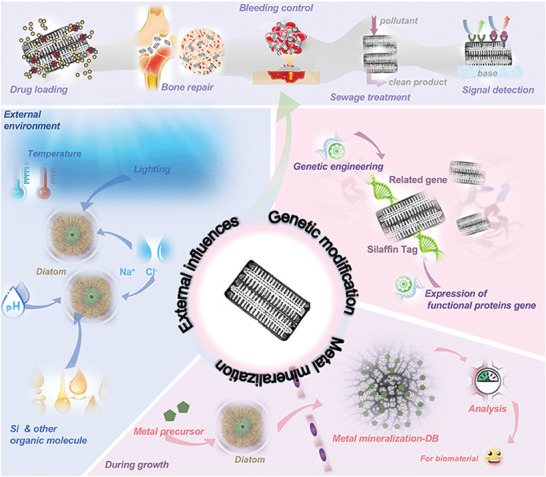
Variations in structure and composition of diatom biosilica (DB) during biofabrication. And the application in the biomaterials field.

In this review, we discuss the biofabrication process of DB, in which the structural adjustability of DB is introduced (**Scheme** **1**). We briefly outline the biological manufacturing process in synthesizing DB and describe the relationship between the structure and properties of DB biomaterials with adjustable properties. We then focus on the structural regulation of DB biomaterials with expandable applications, utilizing diatom biofabrication processes. This will increase research into bioimitated DB biomaterials to support the function of DB, promote innovation in DB as biomedical materials, and further improve and expand the suitability of DB in various applications. This review represents the outstanding progress of DB in various applications of biological materials.

## 2 Biofabrication and Structure

The main component of diatom cell walls is amorphous silica (diatom biosilica, DB), and other organic components include proteins, carbohydrates, etc. DB has a variety of forms, including rounded centric diatoms^[^ ^7^, ^8^ ^]^ and slender *Pennate* diatoms,^[^ ^9^, ^10^ ^]^ ranging in size from 2 µm to 5 mm, with diatoms ranging from 5 to 100 mm most common.^[^ ^11^ ^]^ DB consists of two main components: the gbs and valves (consisting of the epitheca and the hypotheca).^[^ ^10^ ^]^ In general, DB has three structural levels.^[^ ^12^ ^]^ The first layer (cribellum) of the valve is the micron scale, including the gbs and valves; the second structures (cribrum), i.e., the substructures higher than the nanoscale; the third internal surface (areola) is the nanoscale, such as different pore structures. These three levels are integrated to form the structure of DB, the hierarchical micro‐nano‐pore structure.^[^ ^13^ ^]^ Different species of diatoms have a specific pore structure of DB, but they are all formed through the biofabrication process of diatoms silicification.

**Figure 1:**
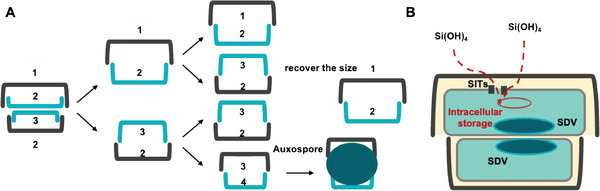
A) Changes in the size of diatom frustules during asexual and sexual reproduction. B) Transport process of silicate.^[^ ^13^ ^]^ (A,B) Reproduced with permission.^[^ ^12^ ^]^ Copyright 2008, American Chemical Society.

The DB biofabrication process of diatom silicification is accompanied by the diatom propagation. Diatoms reproduce through both asexual and sexual reproduction (**Figure** **1**A). During asexual reproduction, the two valves of the parent diatom serve as one valve for each of the two offspring diatoms, resulting in the gradual reduction of the daughter diatom cells. Sexual reproduction allows cells to return to their initial size. Diatoms of different species differ in the timing and flexibility of DB formation.^[^ ^4^, ^10^, ^14^, ^15^, ^16^ ^]^

The biofabrication of DB unfolds as the diatom cycle proceeds, and the diatom cycle depends on the concentration of silicate in the water column (Figure 1B). The diatom actively senses the silicate concentration, initiates the cell cycle and begins to synthesize the DB structure of the cell wall.^[^ ^15^, ^17^, ^18^, ^19^, ^20^ ^]^ Silicates are transported into the cell by silicic acid transport proteins (SITs),^[^ ^4^, ^21^ ^]^ and their transport is regulated by silicate concentrations.^[^ ^12^ ^]^ The concentration of silicates in the cell is higher than that in the water, possibly due to the combination of silicates with certain components to form silicon storage pools.^[^ ^22^ ^]^ Silicates are further polymerized in silicon dioxide deposition bubbles (SDVs), which are the main synthesis site of DB structures.^[^ ^23^, ^24^, ^25^, ^26^ ^]^ The pH and motion state in the SDV affect the synthesis of DB.^[^ ^27^, ^28^ ^]^ The cytoskeletal structure of diatoms, including microtubules and actin, is involved in the movement of SDV, influencing the shape of the SDV and the location of DB synthesis.^[^ ^14^, ^29^, ^30^, ^31^ ^]^ In addition, DB synthesis is regulated by peptides such as proteins silaffins, LCPA, silacidin, and cingulins. These proteins play an important role in silicate polymerization and affect the morphology of the DB structure of diatoms.^[^ ^32^, ^33^, ^34^ ^]^

In summary, the formation of diatom DB is a complex biofabrication process. Through this process, diatom DB is endowed with a unique micro‐nano porous structure, which will help to understand the ecology and function of these microorganisms.

## 3 Properties of DB

The silica structure of DB, formed during the growth and development of diatoms, has a multi‐layered micro and nano pore structure that can adapt to the life activities required by diatoms, thus giving DB unique optical properties, communication, and diffusion with the external environment, as well as mechanical properties that resist external impact, which are fundamental to the subsequent application and development of DB biomaterials (Figure 4).

### 3.1 Mechanical Properties

**Figure 2:**
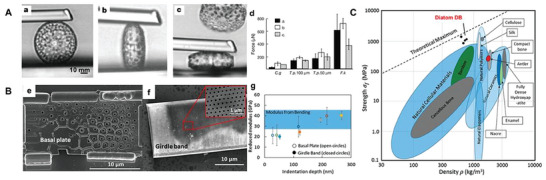
A) Glass needle tests: Live single cells of *T. punctigera* (a–c). d) Forces necessary to break different DB. B‐e) An isolated basal plate and f) a girdle band. g) The reduced modulus as measured from nanoindentation on both an isolated basal plate and girdle band plot against indentation depth. For comparison, the elastic modulus, as determined from bending experiments, is also shown. C) Ashby plot of DB strength versus density for naturally occurring biological materials. The theoretical maximum is determined by extrapolation of the strength and density of diamond. (A) Reproduced with permission.^[^ ^36^ ^]^ Copyright 2003, Springer Nature. (B,C) Reproduced with permission.^[^ ^37^ ^]^ Copyright 2016, National Academy of Science.

Diatoms in nature rely primarily on the structure of DB for defense against natural predators, and the thickness of DB affects the rate of digestion of diatoms by predators: the thicker the DB, the slower the rate of digestion by predators.^[^ ^35^ ^]^ The mechanical protection exhibited by DB is thus dependent on their hardness and cortex, which are comparable to those of medical dental composites.^[^ ^36^ ^]^ Second, the size of DB is inversely proportional to the mechanical forces they can carry; the smaller the area of DB, the greater the external forces they can withstand and the less likely they are to be damaged (**Figure** **2**A).^[^ ^36^ ^]^ In addition, the deformation of DB due to external pressure can be recovered within a certain range, but once they suffer damage, DB will produce brittle deformation rather than plastic deformation.^[^ ^36^ ^]^ The crack propagation process carries out after DB suffers damage. Foramen plays a role in deflecting the crack expansion, while the pore stress propagation along the cribrum layer will fail.^[^ ^37^ ^]^ In the face of external pressure, which is evenly dispersed throughout the DB, the particular structure and material brought about by the valves and gbs can effectively resist external pressure and prevent deformation and damage to the DB.^[^ ^36^ ^]^ In terms of structure, the DB orifice chamber has an I‐shaped structure that can enhance the mechanical properties of the DB. The valves can resist high levels of mechanical stress from 1 to 7 N mm^−2^.^[^ ^38^ ^]^ On the valves, the basal plate mainly carries the mechanical forces, which bending strength can reach 40 GPa (Figure 2B), leading to lower stresses between the holes. Thus the cribrum and areolae layers carry an order of magnitude less stress.^[^ ^37^ ^]^ The gbs have high elasticity modulus and can resist a maximum mechanical stress of 560 N mm^−2^.^[^ ^38^ ^]^ When the gbs are subjected to compressive forces, they can generate large reaction forces.^[^ ^39^ ^]^ In addition, the pores form a silica ring higher than the surrounding structure in response to the concentrated stresses around it.^[^ ^39^ ^]^ In terms of material, the exceptionally high specific strength of DB is due to their honeycomb sandwich panel structure and the extremely low density of biosilica defects.^[^ ^37^ ^]^ DB biomaterials show strengths above other reported materials and are similar to the strongest natural polymers (Figure 2C). The hardness of DB, and their high tolerance to external mechanical forces, allow predators to require a destructive force of approximately 750 N and specially structured teeth to crush DB. Only a few predators, such as copepods and euphausiids, can destroy DB.^[^ ^36^ ^]^

In addition, the purified DB mixed with temperature‐sensitive hydroxybutyl chitosan hydrogels provide anchoring sites for polysaccharide molecules through the interaction anchoring effect of DB. It can improve the mechanical strength of polysaccharide hydrogels after gelling at 37 °C through the intercalation of polysaccharide molecules and hydrogen bonding between polysaccharide molecules and silanol groups on the surface of DB, which will inspire potential applications of subsequent DB materials.^[^ ^40^ ^]^

### 3.2 Optical Properties

**Figure 3:**
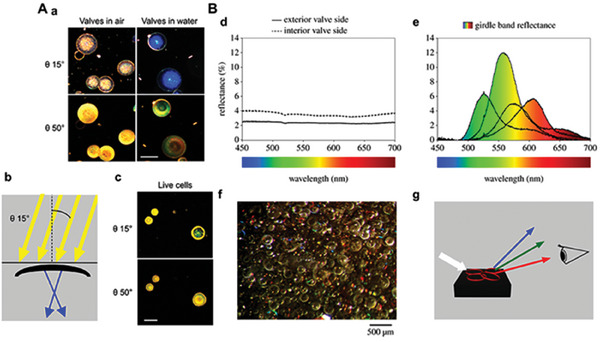
A) Effects from the angle of incidence and refractive index on scattering properties of cleaned valves and living cells. a) Valves in air or water. b) The valve interaction with white light (yellow arrow), showing potential interaction and focusing of blue radiation. c) Scattering properties of live cells. Scale bars, 200 lm. B) Light reflectance on the surface of valves and gbs. d) Reflectance on the exterior and interior side of a valve. e) Reflectance on the gbs at different angles of incidence. f) Photography of valves and gbs in a drop of water was observed at 200× magnification in the light microscope, while incident white light was shone at 308°. Valves appear translucent, but gbs reflected light in alternating colors as a function of the angular location to the incident white light. g) Angular‐dependent transmittance of red, green or blue light on the surface of concentrated frustules in a drop of water was also visible with the naked eye. (A) Reproduced with permission.^[^ ^43^ ^]^ Copyright 2018, New Phytologist. (B) Reproduced with permission.^[^ ^38^ ^]^ Copyright 2011, Royal Society.

The main structures with optical properties in diatom cells are photosynthetic pigments and DB, both of which can regulate the light inside the cell. Among them, DB absorbs, converts, transmits, and reflects the light of different wavelengths, which can effectively defend against UV light and collect photosynthesis‐limited wavelengths, redistribute light inside diatom cells to facilitate photosynthesis, and also prevent cells from being damaged by the light of harmful wavelengths (**Figure** **3**A).

The optical properties of diatom cells are not a simple superposition of the optical properties of DB and the optical properties of other luminescent substances, such as pigments. The emission spectrum of diatom cells has two distinct sets of emission peaks or photoluminescence (PL) peaks. One set of emission peaks is located at 400–500 nm and is the emission peak of DB; the other set of emission peaks is located at 600–700 nm and is the autofluorescence of other luminescent substances, such as pigments within the diatom cell. Because of the low intensity of autofluorescence in diatom cells, researchers call the photoluminescence (PL) of *Thalassiosira pseudonana* diatoms microphotoluminescence (µ‐PL).^[^ ^41^ ^]^ The optical properties of the valves and gbs of the diatom *Coscinodiscus granii* in DB also differ (Figure 3B). The valves attenuate light at shorter wavelengths, whereas the gbs attenuate at longer wavelengths and can refract iridescent light depending on the direction of the incident light.^[^ ^38^ ^]^ The valves of DB have a light‐limiting effect, where incident light is restricted by the regular pore pattern on the surface of the DB to a few microns of speckle, the size of which depends on the wavelength of the light, an effect attributed to the superposition of wavefronts from diffraction at the edge of the pores of the DB, and such effect does not occur in the UV region.^[^ ^42^ ^]^

The promotion of photosynthesis by DB is manifested in two main ways. The first is the distribution of photosynthetically active light within the cell through transmission. During photosynthesis in diatom cells, DB and chloroplasts are optically coupled to exhibit an efficient light‐trapping mechanism that prevents the backscattering of transmitted light and thus enhances light absorption,^[^ ^43^ ^]^ which is caused by the strong asymmetry between the cribrum and foramen cycle structures.^[^ ^44^ ^]^ Second, DB and chloroplasts convert harmful UV light into light required for photosynthesis through photoluminescence. DB can convert harmful UV light into blue light essential for photosynthesis through photoluminescence. The conversion of DB at this UV wavelength is more efficient than other emission jumps within the visible spectrum.

**Figure 4:**
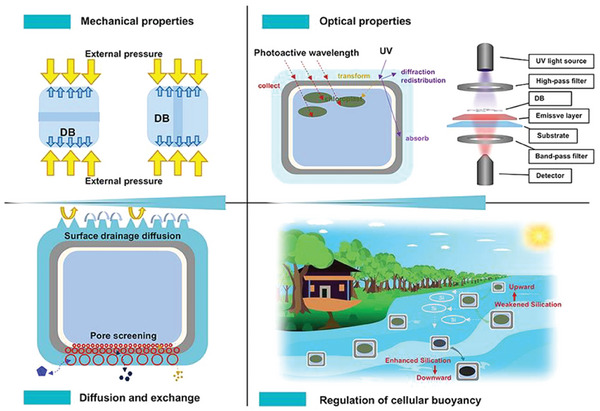
Depending on the DB, its mechanical properties, optical properties, diffusion and exchange, and regulating cell buoyancy. Partial content Reproduced with permission.^[^ ^47^ ^]^ Copyright 2018, Springer Nature.

UV light will damage the DNA of diatom cells and cause DNA degradation. DB protects diatom cells from UV damage mainly through three mechanisms: absorption of UV by amorphous silica and organic residues, conversion of UV to photosynthetically effective wavelengths through photoluminescence, and distribution of UV outside diatom cells through transmission and diffraction (**Figure** **4**).^[^ ^45^ ^]^ The DB of different diatom species projects UV light differently.^[^ ^46^ ^]^ Moreover, UV redistribution caused by the silica valves is an essential evolutionary reason for the existence and evolution of the DB, and the facilitation of photosynthesis and protection of genetic material by DB may be sufficient to help explain the surprisingly large number of diatom species with different geometries produced by convergent evolution.^[^ ^47^ ^]^ The UV screening effect of DB makes it useful for preventing the degradation of polymers and paints and has the potential for novel applications such as producing biocompatible sunscreens.^[^ ^45^ ^]^

### 3.3 Functions of Diffusion and Exchange

Between diatom cells and the surrounding seawater, the multisized pores of DB play a screening role in the diffusive exchange of gases, nutrients, and other metabolites. The porous structure of DB is selective in nutrient or particle transport, and the surface micromorphology of DB influences the diffusion and convection of submicron particles.^[^ ^48^, ^49^ ^]^ When particle flow is present on the surface of DB, the micromorphology of DB controls the diffusion and convection of particles on their surface. Whereas in the absence of flow, the micromorphology of DB reduces the diffusion of particles. Under the divergent diffusion effect of DB, different shell microstructures have different effects on the diffusion and convection of Brownian particles, such as the higher local particle concentration in the bulge region around areolae.^[^ ^48^ ^]^ DB classifies particles during the control of particle diffusion and convection, acting as a selector for particles that can reach the cell membrane and its receptors (Figure 4). This role is important in the nutrient uptake of colloids and particles into the cell, especially when the particles are much smaller than areolae, and can help increase nutrient uptake by reducing areolae blockage. There is a potential role for chemical communication and nutrient exchange between cells and the environment, gas exchange, and virus control.

### 3.4 Regulating Cell Buoyancy

Changes in the density of DB can alter the buoyancy of cells. Diatoms can change the sinking rate by increasing the density of DB through silicification or by regulating the content of protoplasmic solutes (Figure 4), and the two modifications interact to change the buoyancy of diatom cells. And regulating the density of protoplasm can counteract the effect of silicification, so the buoyancy of diatom cells can be offset by adjusting the protoplasm density. When diatoms are under suboptimal growth, the degree of silicification increases and the density of protoplasts decreases due to abiotic conditions, which reduces the buoyancy and makes diatom cells move to the location of optimal growth conditions. Thus regulating the growth state of diatom cells. In addition, diatoms also enhance the sinking of parasite‐infected cells in the population through silicification to eliminate these cells.^[^ ^49^ ^]^

## 4 Purification Procedure of DB

Before using DB as a biomedical material, to obtain the inorganic DB in diatom cells, it is necessary to remove most of the organic matter, carbohydrate and so on. At present, there are three common methods for processing purified DB. i) To purify DB from diatom cells, first were filtered, washed with deionized water, and then treated with a mixture of HCl and H_2_O_2_ to remove organic matter. After repeated processing and thorough rinsing, the purified DB were vacuum‐dried and stored.^[^ ^50^, ^51^ ^]^ ii)Followed by the collection of algal mud, a lye solution of NaOH and urea was mixed with algal mud, washed, and frozen. The treated slurry was added to a piranha (a mixture of sulfuric acid and H_2_O_2_) solution at 75 °C, stirred, and pH adjusted to neutral to obtain DB.^[^ ^52^ ^]^ iii) Collected diatom cells were washed and treated with sulfuric acid and nitric acid at 60 °C. The resulting DB were washed with water and ethanol before being dried in a vacuum drier at 60 °C for 12 h.^[^ ^53^, ^54^ ^]^ It can be selected according to the experimental requirements and the characteristics of diatom samples. No matter which method is chosen, needs to be taken to take proper safety measures when handling diatom samples.

## 5 Regulating DB through Biofabrication and Its Application

The micro‐nanoporous structure and morphological changes of DB are the prerequisites for their application as biomaterials. The structure and morphology can be changed by changing illumination, temperature, and other factors, including pore size, particle size, and surface state, as well as the selective expression of genes (**Table** **1**). Considering the diversity of DB structure and function, various ways, including chemical modification, biological modification, and gene regulation, can be used to achieve the purpose of the regulation. Diatoms can currently be propagated in the laboratory in a controlled manner, manipulated both externally and genetically. Both types of manipulation have their advantages. The external manipulation does not involve altered genetic modifications, including factors such as growth environment, metal elements, and organic molecules affecting the structure of the diatom cell wall. On the other hand, genetic manipulation of DB synthesis establishes an association between the relevant genes and structure formation, which can be further exploited to alter the structure of DB.

**Table 1:** Summary of structural changes of DB by changing external conditions.

External conditions	Main parameters	Effect on DB	Diatom species & references
Lighting	Wavelength, intensity of light	Change the average diameter of DB and the average diameter of pores; Affect the silica content and thickness of DB.	*Coscinodiscus granii* ^[^ ^55^, ^56^ ^]^
Salinity	Seawater is dominated by Na^+^ and Cl^−^	Related to the species of diatoms; Change the DB silicification, change the pore structure, resulting in the change of DB symmetry.	*Thalassiosira weissflogii* ^[^ ^60^ ^]^
Temperature	Low or high temperature	It is related to the species of diatom and the content of silica. Change the size of DB and the shape of DB.	*Ditylum Brightwellii*(West)Grunow^[^ ^59^ ^]^
pH	Over acid or base	Both over‐acid and over‐base can cause DB to thicken and lead to changes in pore structure.	*Thalassiosira weissflogii* ^[^ ^57^ ^]^
Silicate and other nutrients	Large influence elements such as silica, micro nutrient elements such as phosphate, etc.	The content of silica affects the texture and pattern of DB. The type of silica affects the composition and micro‐nano structure of DB. The concentration of phosphate affects the particle size of DB.	*Thalassiosira pseudonana* ^[^ ^61^ ^]^ *Thalassiosira weissflogii* ^[^ ^58^ ^]^
Biological parameter	Biological inhibitors such as colchicine and paclitaxel	The micro‐nanostructure of DB is affected, such as cleavage furrows and spines.	*Aulacoseira islandica* ^[^ ^62^ ^]^
	bacteria and predators, etc	The influence is complex, so it is not suitable for modifying DB.	∖

### 5.1 External Influences

The method that the structure and morphology of DB change with the change of culture conditions can be applied to preparing DB biomaterials as the main method or auxiliary means. The periodic structure and pore diameter of DB are two of the most important parameters in the DB application, and illumination, temperature, and salinity are abiotic parameters that mainly affect these.^[^ ^55^ ^]^ It is simple to change the structure of DB by changing culture conditions such as light, temperature, or salinity during the growth process. Long‐term cultivation of diatoms can realize morphological changes and produce controllable variations in the structure of DB, indicating that DB has phenotype plasticity.

#### 5.1.1 Lighting

Colored light and high‐intensity light are survival pressure for diatoms. Changing the light condition in diatom culture can not only change the shape, thickness, pore size, and pore density of DB, but also change the growth rate of diatoms and the silica content of DB. The main factor affecting the structure of DB is the wavelength and intensity of light.^[^ ^55^, ^56^ ^]^ They are correlated with the structure of DB.

**Figure 5:**
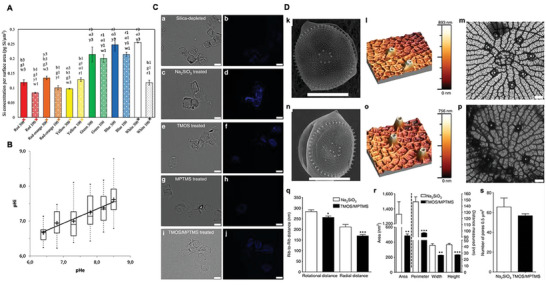
A) Si concentration per surface area of *C. granii* grown under different light wavelengths at 100 and 300 µmol photons m^−2^ s^−1^. The + below indicates that there were significant differences (*p* ≤ 0.05) between the different irradiance of the same wavelength. B) Influence of external pH on intracellular pH. C) DB synthesis occurs when *T. weissflogii* is cultured in the presence of silica precursor. Brightfield a,c,e,g,i) and fluorescent b,d,f,h,j) images of *T. weissflogii*. Scale bar, 10 µm. D) SEM micrographs of *T. weissflogii* and TMOS/MPTMS *T. weissflogii*. Surface topography of the valve surface. *T. weissflogii* illustrate the decreased pore parameters in the modified diatom. Distance between ribs decreases in TMOS/MPTMS *T. weissflogii*. Architectural properties of the pores on the valve surface are reduced in TMOS/MPTMS *T. weissflogii*. The density of pores on the valve surface is unaltered (k‐s). (A) Reproduced with permission.^[^ ^55^ ^]^ Copyright 2018, Elsevier. (B) Reproduced with permission.^[^ ^57^ ^]^ Copyright 2012, PLOS ONE. (C,D) Reproduced with permission.^[^ ^58^ ^]^ Copyright 2013, Springer Nature.

White, blue, green, red, orange, and yellow lights at high (300 µmol photons m^−2^ s^−1^) and low (100 µmol photons m^−2^ s^−1^) light intensities, respectively, affect the shape, thickness, and micro‐nanostructure of DB, and cell growth rate of *Coscinodiscus granii* diatoms. The nanostructure of DB is significantly affected by red, yellow, green, and blue lights at high light intensity. At low light intensity, green lights cause the average diameter of DB and pore diameter to decrease significantly (**Figure** **5**A). These structural changes are sufficient to alter the original photonics properties of DB.^[^ ^55^ ^]^ The silica content of DB is related to the thickness rather than to the species and cell size of diatoms.^[^ ^56^ ^]^ Except for yellow light, the higher the intensity of the other five wavelengths, the higher the silica content per unit area of DB, and the smaller the flat surface area. At the same light intensity, blue lights make DB have the highest silica content.^[^ ^56^ ^]^ In terms of growth rate, there is no significant difference in the growth rate of diatoms under the action of the six wavelengths. However, under low light intensity, the growth rates of diatoms under yellow, red, orange, and green lights are significantly lower than that under blue and white lights.^[^ ^55^ ^]^

#### 5.1.2 Salinity

Salinity is a measure of the total dissolved inorganic matter in a liquid, which in seawater is mainly Na^+^ and Cl^−^.^[^ ^59^ ^]^ Changes in salinity can cause changes in the morphology of DB by affecting the photosynthesis of diatoms and their silica metabolism of diatoms, but have no significant effect on the accumulation and incorporation of silicate.^[^ ^60^ ^]^ Increasing salinity slows down the silicification of DB and leads to larger pores in DB, while changes in salinity can cause symmetry changes in DB.^[^ ^60^ ^]^ In addition, the effect of salinity on the structure of DB is related to diatom species.^[^ ^59^ ^]^ The diatom *Thalassiosira weissflogii* showed structural adjustments in DB adapted to changes in salinity under low salinity culture conditions, with an increase in ellipsoidal DB and changes in the distribution and location of specific silica structures.

#### 5.1.3 Temperature

The effect of temperature on diatoms is related to diatom species, and different species of diatoms respond differently to changes in temperature in terms of cell size, with some decreasing in size associated with decreasing temperature, while others have no apparent temperature responsiveness in cell size. In Korean coastal waters, marine diatom *Ditylum brightwellii (West) Grunow* shows an increase in prismatic cells at low temperatures and the predominance of cylindrical shapes at high temperatures.^[^ ^59^ ^]^ Other environmental factors did not significantly affect the size or shape of either cell. This dramatic change in diatom size and shape with temperature suggests that changing temperature can alter diatom shape and size.

#### 5.1.4 pH

Organelle within diatom cells require a specific pH environment to function and carry out certain physiological processes. The internal pH of SDV, the organelle in which diatom silicification takes place, must be acidic. Changes in the pH of the diatom growth environment and the pH inside the diatom cell are positively correlated, meaning that changes in the external pH level will affect the acid‐base balance inside SDV, which will affect the swelling of SDV and thus the formation of DB.^[^ ^59^ ^]^ When the external pH changes, the porosity and pore size of DB are affected, showing a maximum change at a specific pH and a decrease with acidification or alkalinization of the environment. On the other hand, any unsuitable pH reduces the growth rate of diatom cells and increases the growth cycle, allowing diatom cells to take in more silicate and synthesize more biosilica during growth, ultimately leading to thicker DB. *Thalassiosira weissflogii* was incubated in different cultures with pH ranging from 6.5 to 8.4. It was found that the growth rate was greatest at pH 7.8, where more acidic and more alkaline environments both led to a decrease in growth rate, thickening of DB, and a change in pore parameters (Figure 5B).^[^ ^57^ ^]^

#### 5.1.5 Silicate and Other Nutrients

Silicate, as the main source of DB, directly affects the quality of DB. When there is insufficient silica in the environment and superfluous other nutrients, diatoms will undergo adaptive changes, slowing down the rate of cell division, weakening the mineralization process of DB, and resulting in thinner DB. Abiotic parameters also interact with each other to influence the structure of DB. Temperature and silica salt content have an interrelated effect on the morphology of DB. In the culture of the diatom *Thalassiosira pseudonana*, the effect of temperature on the cell growth is related to the stage of cell growth, by increasing cell density during the silica‐rich phase at high temperatures. While after the diatom plateau phase, when the diatom population continues to divide using dissolved silica from dead diatom cells, lower temperatures increase the diatom cell density. The DB of *T. pseudonana* is divided into two patterns: reticulate and dendrite, with the latter having a sparser texture than the former. When silica is sufficient, the temperature does not significantly affect the DB pattern. However, under silica‐limited conditions, the higher the temperature, the higher proportion of the reticulated DB.^[^ ^61^ ^]^

Different silicates with different elemental ratios can have different effects on the state of DB (Figure 5C,D). Lang et al. set up an experimental group using alkoxysilane tetramethoxysilane (TMOS) and 3‐mercaptopropyltrimethoxysilane (MPTMS) as the source of silica during the growth of *T. weissflogii*, and conventional sodium silicate as the control group. Diatom DB synthesis is not observed in silica‐depleted groups or experimental group. The silica precursor capable of generating a siloxane backbone is necessary for DB synthesis. The overall morphology of the experimental DB was unchanged, but the chemical composition and shell sheet structure changed, with the DB having a reduced silica content and a higher sulfur and carbon content than the control group DB. Also, the main form of sulfur in the experimental groups was the thiol group, whereas the thiol fraction was not present in the control group. The reduction in silica density and the incorporation of thiol groups both led to a reduction in the mechanical strength of DB. In terms of structure, the radial distance between ribs and the distance in the rotational direction shortened in the experimental group, with a subsequent significant reduction in pore size, which can be attributed to changes in the siloxane skeleton of the DB following the incorporation of thiol groups.^[^ ^58^ ^]^

In addition to macro nutrient elements such as silica, trace elements such as nitrogen and phosphorus, and vitamins can also cause changes in the morphology of DB. For example, as the phosphate concentration increases from a few × 10^−6^ m to 100 × 10^−3^ m, the diameter of the DB increases from 50 to 600 nm. Phosphate may act as a cross‐linking agent to establish hydrogen bonds and electrostatic interactions, thereby precipitating the silica. Similarly, the concentration of multivalent anions (e.g., citrate and sulphate) also alters the morphology of the shell.^[^ ^59^ ^]^

#### 5.1.6 Biological Parameter

Parameters affecting the structure and surface state of DB include biological parameters, i.e., silica and other nutrient salts and organic molecules, and abiotic parameters. Through the influence of these factors in various aspects of DB, the regulation of the micro‐ and nanostructure and surface morphology of DB can be achieved, expanding the prospects of DB in various applications, including drug delivery, bone tissue materials, hemostat agents, immobilized enzymes, sensor detection, and contaminant treatment.

**Figure 6:**
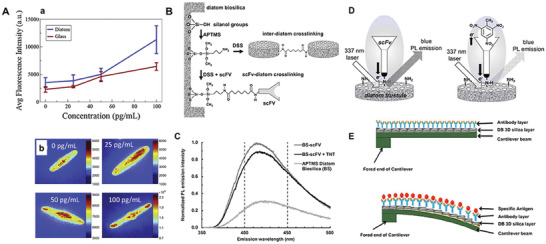
A‐a) A comparison plot of average fluorescence intensity on diatom biosilica and glass. b) Representative fluorescence images of the frustules. B) Schematic of biosilica functionalization by scFv. C) Comparison of PL emission spectrum from APTMS‐functionalized Pinnularia diatom biosilica frustules (BS), scFv antibody functionalized diatom biosilica crosslinked with disuccinimidyl suberate (BS‐scFv), and scFv‐functionalized diatom biosilica after immunocomplex formation with 4.8 × 10^−7^ m 2,4,6‐trinitrotoluene (BS‐scFv þ TNT). All spectra were normalized relative to scFv‐functionalized diatom biosilica. D) Conceptual diagram of photoluminescence quenching upon binding of TNT with the scFv‐functionalized diatom frustule. E) Schematic representation of 3D siliceous microcantilever‐based biosensor a resting state and b sensing state. (A) Reproduced with permission.^[^ ^64^ ^]^ Copyright 2019, Elsevier. (B–D) Reproduced with permission.^[^ ^65^ ^]^ Copyright 2016, Elsevier. (E) Reproduced with permission.^[^ ^66^ ^]^ Copyright 2020, Springer Nature.

Previous research on DB using organic molecules has focused on applying organic fluorescent dyes to DB to investigate the mechanism of the formation of DB biosilica. The current demand for new nanomaterials has led to a shift in thinking. Researchers have started using organic molecules involved in the mineralization of DB or combined with DB to prepare new nanobiomaterials, changing the structural surface shape of DB biosilica materials and extending their application directions. DB biosilica with photoluminescence effect can be used as optical nanomaterials when doped with different fluorescent dyes (**Figure** **6**A); as drug delivery and release systems and bone tissue medical materials when doped with different drugs; and as immobilized enzyme systems and biomonitoring sensing platforms when doped with different peptides and enzymes (**Table** **2**).

**Table 2:** Summary of organic molecular influence on DB.

Organic molecule	Effect on diatom cells	The distribution of organic molecules on DB	Applicable direction	Diatom species & references
Rhodamine	Rhodamine with amino substituents did not show cytotoxicity, while Rhodamine with different carboxyl substituents showed greater cytotoxicity to diatom cells.	It is embedded in DB.	The PL property of it is a superposition of DB and Rhodamine. It can be used in optical materials	*Coscinodiscus granii, Coscinodiscus wailesii* ^[^ ^87^ ^]^
Rhodamine B and PDMPO	The diatom cells grew normally at appropriate dye concentrations.	It is embedded in DB.	PDMPO and Rhodamine B constitute the Forster transfer pair, which can be used as a self‐replicating microlaser with its own resonator.	*Cyclotella meneghiniana* ^[^ ^63^ ^]^
PE‐Syl	There was no obvious cytotoxicity and the diatom cells could divide and proliferate normally.	It is embedded in DB.	The incorporation of PE‐Syl can adjust the PL properties of DB, and the PL properties of the composite materials obtained are different from those of DB and PE‐Syl.	*Thalassiosira weissflogii* ^[^ ^3^ ^]^
Iridium complex	Does not destroy the diatom cell physiological activity; diatom cells divide normally.	Form a phosphorescent silica cluster with a diameter of less than 100 nm, consisting mainly of residual organic matter and quasi‐spherical DB NPs with a diameter of less than 10 nm.	Potential applications in imaging, sensing and biomedical photonics.	*Thalassiosira weissflogii* ^[^ ^88^ ^]^
Alendronate	Diatom cells grow normally	The drug is incorporated into DB.	Used for drug loading, and bone tissue repair material.	*Thalassiosira weissflogii* ^[^ ^71^ ^]^

When organic fluorescent dyes are embedded in the DB structure, the DB has a protective effect on the fluorescent dyes and can prevent dye degradation. When some fluorescent dyes are doped into DB, the PL spectra of the materials obtained are a simple superposition of the spectra of the fluorescent dyes and the DB. The fluorescence emission intensity is dependent on the fuel concentration. While some other fluorescent dyes, when doped into DB, give PL spectra of the composites distinct from either the spectra of the diatoms or of the dyes. In addition to doping a single fluorescent dye, some researchers have doped two dyes with interacting properties into DB simultaneously, e.g., PDMPO and Rhodamine B, into DB of diatom *Cyclotella meneghiniana*.^[^ ^63^ ^]^ Through biofabrication, PDMPO and Rhodamine B form a Förster transfer pair, PDMPO as the donor and Rhodamine B as the acceptor. The FRET efficiency of PDMPO and Rhodamine B in DB was found to be 0.25 ± 0.12, and the average fluorescence lifetime of the donor was reduced by 0.75× due to the presence of the acceptor. This novel hybrid biomaterial forms a Förster transport system that may lower the threshold for stimulated emission, forming a self‐replicating microlaser emitter carrying its own resonator. If the fluorescent dye contains silica groups, it makes incorporation into DB easier and covalently binding to DB possible. Ragni et al. prepared DB biomaterials that deeply penetrate and incorporate the dye into the core of the DB silica shell. This process distributes and protects the dye far beyond the algal skeleton's functionality. Progressive biomaterials retain the original micro‐nanolayered porous structure of DB and generate discontinuous random laser action by exciting fluorescent dye luminescence. This gives it the potential to be used in the preparation of noncoherent random lasers.^[^ ^3^ ^]^

**Figure 7:**
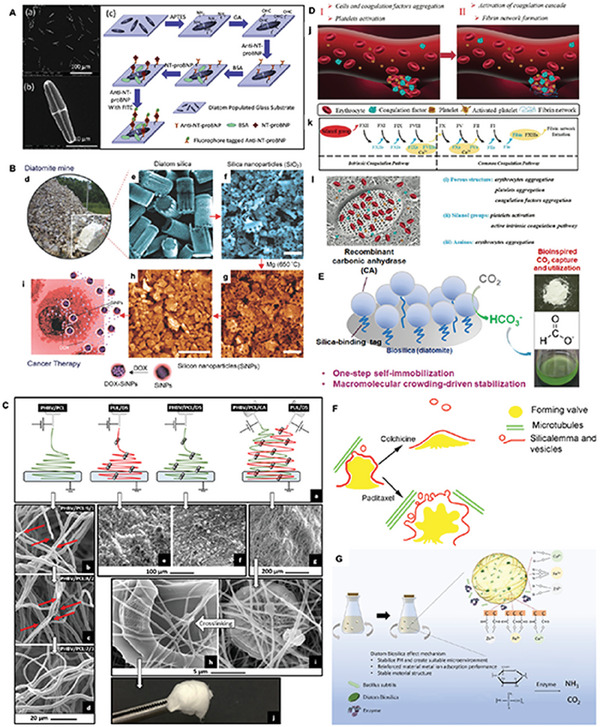
A‐a) SEM image of diatom frustule‐populated glass slide and b) zoomed in on a single frustule. c) Schematic view of diatom‐based immunoassay for NT‐proBNP detection. B) The concept from the mine to cancer therapy: natural and biodegradable theranostic silica nanocarriers from diatoms for sustained delivery of chemotherapeutics. C) Schematic illustration of conventional wet electrospinning and wet co‐electrospinning scaffold fabrication systems. D) Formation of clot at the site of vascular wound after applying CDDs‐TBA. E) Stability‐controllable self‐immobilization of carbonic anhydrase fused with a silica‐binding Tag onto DB for enzymatic CO_2_ capture and utilization. F) Scheme of possible mechanism of microtubule inhibitor influence on the spine formation on the valve. G) Mechanism diagram of wastewater treatment by probiotics with DB. (A) Reproduced with permission.^[^ ^64^ ^]^ Copyright 2019, Elsevier. (B) Reproduced with permission.^[^ ^79^ ^]^ Copyright 2023, Elsevier. (C) Reproduced with permission.^[^ ^74^ ^]^ Copyright 2019, Elsevier. (D) Reproduced with permission.^[^ ^80^ ^]^ Copyright 2020, John Wiley and Sons. (E) Reproduced with permission.^[^ ^77^ ^]^ Copyright 2020, American Chemical Society. (F) Reproduced with permission.^[^ ^62^ ^]^ Copyright 2018, The Company of Biologists. (G) Reproduced with permission.^[^ ^52^ ^]^ Copyright 2021, Elsevier.

Due to DB's high specific surface area, high porosity, SERS sensitivity, PL effect (Figure 6B–D), and the presence of silanol groups, DB can be used as potential protein adsorbents and can be connected to chemical groups as detection sensors. The main approaches to obtain DB biosensors are 1) Firmly binding proteins to DB to achieve good reproducibility and stability of captured target molecules in immune monitoring.^[^ ^67^ ^]^ 2) Using DB's enhancement of Raman and fluorescent signals to obtain a fluorescent imaging immunoassay platform (**Figure** **7**A).^[^ ^64^ ^]^ 3) Using the photoluminescence effect to detect substances by bursting or enhancing the PL effect of DB.^[^ ^65^ ^]^ 4) Using DB to play an auxiliary role in other sensors to improve the detection effect of the sensors (Figure 6E).^[^ ^66^, ^68^ ^]^

DB has strong potential for drug delivery applications to prolong drug release and enhance therapeutic effects (Figure 7B). The main ways to encapsulate drugs for drug delivery by DB are 1) obtaining nanoscale Si NPs by mechanical fragmentary DB multi‐layered structure for drug delivery.^[^ ^69^ ^]^ 2) Selecting functional macromolecules, such as chitosan, to surface functionalize DB silica and load the drug for drug delivery.^[^ ^70^ ^]^ 3) Adding the drug to diatom media and mineralizing the drug onto DB through the biofabrication process of DB synthesis.^[^ ^71^ ^]^ The high surface area and high porosity of DB make it have great application potential in drug delivery, which can reduce the toxicity of drugs, prolong the release time of drugs, and enhance the therapeutic effect. Some researchers mineralized the drug onto DB by adding it to the diatom culture medium and utilizing the biofabrication process of DB synthesis. Cicco et al. successfully obtained Na ALE functionalized DB materials by mineralizing alendronic acid, a clinical therapeutic drug for bone metabolic disorders, onto DB through biofabrication process. The loading percentage of Na ALE on DB is 1.45% w/w, and this Na ALE DB material can promote the growth of osteoblast‐like cells (SaOS‐2) and bone marrow stem cells (BMSC), and effectively inhibit the growth of anti‐osteoclast. It has good ability of osteo conductive and activation of tissue regeneration model.^[^ ^71^ ^]^

Silica plays a vital role in bone formation and maintenance, improving osteoblast function and inducing mineralization. Bone deformation and long bone deformities are usually associated with silica deficiency. The cytocompatibility of diatomic biosilica and its unique multilayer porous structure can achieve mechanical interlocking of cell adhesion, giving the material a great capacity for cell growth and bone mineralization. Therefore, DB is widely used as a bone tissue repair material. The main ways to use DB as bone tissue repair material are 1) flake DB is directly used as a platform for osteoblast growth.^[^ ^72^ ^]^ 2) DB is added to bone tissue scaffolds as silica supplements to improve the osteoinductive properties of bone tissue scaffolds.^[^ ^73^ ^]^ 3) DB are doped into nanofibers using electrostatic spinning technology to obtain a multifunctional biogenic 3D fibrous scaffold with good biocompatibility for bone tissue (Figure 7C).^[^ ^74^ ^]^ 4) DB continuum is obtained by calcinating pure DB, and the resulting 3D cell growth platform could facilitate bone grafting and other possible 3D cell growth techniques.^[^ ^75^ ^]^

To establish effective cascade reactions for technical applications, through multiple enzyme‐catalyzed steps (cascade reactions), the cellular metabolic pathway rapidly converts molecules into useful products without creating waste. This pathway primarily focuses on controlling the location of the enzyme by immobilizing it in custom compartments, thus imitating the high degree of organization in natural organisms.^[^ ^76^ ^]^ Immobilization improves the stability and reusability of the enzyme (Figure 7E).^[^ ^77^ ^]^ Synthetic mesoporous silica materials are widely used as carriers for enzyme immobilization due to their high enzyme loading capacity and the excellent mass transfer properties of their mesoporous interconnected channels. In contrast, the enzyme‐supporting properties of natural DB silica materials have only been carried out in recent years.^[^ ^78^ ^]^ It is mainly through binding catalytic enzymes to protein tags, which are then immobilized on the surface of DB, to form an artificial enzyme cascade catalytic system.^[^ ^76^ ^]^

The value of DB in emergency hemostat materials was first reported by our team internationally in 2015. It was found that DB obtained from artificial culture have a unique micro‐ and nanolayered porous structure, which not only accelerates platelet hemostat thrombus formation, but also activates endogenous clotting pathways and forms stable clots, and is superior to Quikclot (the most advanced American commercial zeolite hemostat agent) in terms of hemostat speed, secondary bleeding volume, total bleeding volume, and side effects.^[^ ^50^ ^]^ Subsequently, the hemostat performance of three typical central diatom DB were compared, it was found that the structural characteristics of DB determine their procoagulant activity, and DB with larger specific surface area and water absorption rate have faster hemostat.^[^ ^54^ ^]^ After this report, it received positive evaluation and follow‐up studies from international experts. Han et al. found that the rate of blood clot formation of DB was negatively correlated with size and pore size, and the strength of blood clot was also negative correlation with the specific surface area of it through Pearson correlation analysis.^[^ ^53^ ^]^ In 2020, Lee et al. followed up our work and found that the unique micro‐nano structure of DB makes them exhibit extreme hemophilicity.^[^ ^81^ ^]^

**Figure 8:**
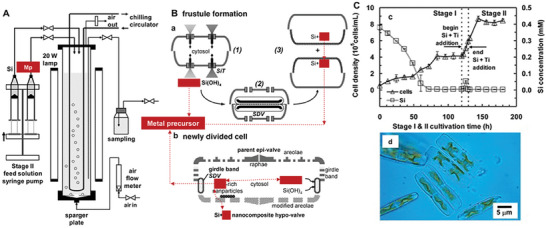
A) Photobioreactor for the cultivation of the diatom under controlled delivery of soluble silicon and metal precursor. B) A conceptual model for metabolic insertion metal into diatom DB. a) Cellular processes: (1) surge uptake of Si and metal (SiT = Si transporter); (2) valve development; (3) cell division; b) DB from a newly‐divided cell from Stage II of cultivation, showing intracellular metal located in the new hypo‐valve, metal‐rich nanoparticles, the developing girdle band, and the cell cytosol. C) Bioreactor cultivation situation of *Pinnularia sp*. cells. c) Cell number density and dissolved silicon concentration versus time for Stages I and II of bioreactor cultivation. d) light micrograph of living diatom cells at the end of Stage I, just before the addition of titanium to the cultivation medium. (A,B) Reproduced with permission.^[^ ^92^ ^]^ Copyright 2008, Elsevier. (C) Reproduced with permission.^[^ ^97^ ^]^ Copyright 2008, American Chemical Society.

On the basis, we has developed several new dosage forms of DB hemostat materials, including: hemostat powder,^[^ ^82^ ^]^ hemostat sponge,^[^ ^83^, ^84^ ^]^ hemostat aerogel (Figure 7D),^[^ ^80^ ^]^ hemostat granules,^[^ ^85^ ^]^ hemostat gauze,^[^ ^86^ ^]^ etc. The above studies proved the great potential of DB as emergency hemostat materials and elucidated to some extent the importance of DB micro‐nano structures in the pro‐coagulation process. The main methods to achieve rapid hemostat of DB are 1) blending with functional macromolecules to prepare hemostat agents with different dosage forms to enhance the hemostat performance while adapting to different bleeding scenarios.^[^ ^81^ ^]^ 2) Selecting calcination conditions or different types of DB. 3) Binding with pro‐coagulant components, such as thrombin and Ca^2+^ to accelerate the coagulation process (**Figure** **8**B).^[^ ^51^ ^, ]^ 4) Using tert‐butanol to create pores after co‐blending with macromolecular polysaccharides to restore the porous structure of closed DB and to improve the hemolysis of DB.^[^ ^53^, ^54^ ^]^

The addition of inhibitors also affects the morphology and structure of DB. Bedoshvili et al. have studied the effects of the microtubule inhibitors colchicine and paclitaxel on the shell structure of the centromeric diatom *Aulacoseira islandica* (Figure 7F).^[^ ^62^ ^]^ Colchicine blocked the polymerization of microtubule proteins and inhibited the formation of linkage spines, causing the cleavage furrow to be blocked, forming a single lateral shell sheet on the side of cylindrical DB and eventually a single hollow tube. In contrast, Paclitaxel is an inhibitor of microtubule depolymerization and allows spines to form complex branching shapes. Biological factors such as bacteria or other microorganisms may also influence DB's morphological changes. However, methods that use natural enemies of diatoms to alter the structural morphology of DB are more difficult to manipulate due to the high sensitivity and complexity between different organisms, such as diatoms and predators.^[^ ^59^ ^]^ In addition, DB, combined with microorganisms, is applied to water pollution treatment, by promoting bacterial growth, maintaining and prolonging overall activity and stability, and enhancing the adsorption of metal ions. It is effective in degrading organic pollutants, inorganic pollutants NH_4_, and metal ions Fe^3+^, Zn^2+^, and Cu^2+^ (Figure 7G).^[^ ^52^ ^]^

### 5.2 Metal Mineralization

The direct addition of some suitable metal precursors to the culture medium can mineralize the metals onto DB using the biofabrication process. To maximize the amount of metal mineralized onto DB, culture is often carried out in a photobioreactor, which controls the addition rate of the culture medium and prevents the precursors from precipitating in the medium. In addition to using a photobioreactor, suitable metal precursors can be selected to improve the mineralization effect. The requirements for metal precursors are that they do not cause serious effects on diatom growth at specific concentrations, do not precipitate in the medium, can be taken up by diatom cells, and have a high incorporation rate on DB. In addition, mineralization can be improved by screening diatom species resistant to mineralized metal elements.

Mineralization of metallic elements or organic molecules to DB is often carried out using a two‐stage culture method. The first stage is a normal diatom culture, which aims to deplete the silica in the culture medium and requires diatom cells to reach the plateau stage without dividing within one light‐dark cycle so that the cell cycles of all cells in the diatom population are synchronized. In the second phase, metal precursors are added to allow the metal elements to participate in forming DB and eventually mineralize into the DB structure. In the second stage, diatoms can be cultured using the starvation method, where only a small amount of silicate is added to ensure limited diatom division, while a higher amount of metal precursors are added to the medium to force diatom cells to take up large amounts of the target metal elements. The mineralization of metallic elements into DB is usually followed by high‐temperature thermal annealing, which transforms the amorphous metallic compounds on the DB into regular structures with a crystalline shape (Figure 8A).

Not all metallic elements can be mineralized onto DB by bioculture. It is known that germanium, titanium, calcium, iron, zinc, aluminum, and gold can be mineralized onto DB by bioculture or deposited on the surface of DB (**Table** **3**).^[^ ^89^ ^]^ Among these, titanium and calcium are elements existing in DB, iron and zinc are trace elements required for diatom growth, and germanium is a homologous neighboring element similar to silicon. However, excessive amounts of these elements can affect the viability of diatom cells. Whereas ions of other elements such as nickel, cadmium, copper, and silver are not bound to the DB biological silica structure. They are more likely to deform the DB by causing survival stress to the diatoms, or they enter the diatom cells and trigger the detoxification mechanism of the diatom cells, forming metal nanoparticles in the presence of phytochelatins (chelating elements) in the diatom cells.^[^ ^90^, ^91^ ^]^

**Table 3:** Summary of biomineralized DB with metal elements.

Metallic element	Common precursors	Influence on DB structure	Mineralized amount	Chemical composition	Distribution on DB	Application direction	Diatom species & references
Ge	Soluble germanium, germanium compounds such as Ge(OH)_2_	The overall shape of the DB is intact, but the micro and nano structures are affected, such as forming special nano comb structures, changing pore size and shape, and blocking pore structures	0.24–0.97 wt%	Forming a compound of germanium silicon or an oxide of germanium	The pores of DB are filled with germanium nanoparticles, and the germanium nanoparticles are evenly embedded in DB.	With the properties of electroluminescence and photoluminescence, it can be used in semiconductor materials and microelectronics devices.	*Pinnularia sp*.^[^ ^92^, ^95^, ^96^ ^]^ *Nitzschia frustulum* ^[^ ^93^, ^94^ ^]^
Ti	Ti‐HCl,Ti(OH)_4_,TiOSO_4_,Ti‐BaldH, etc.	A proper amount of titanium does not affect the structure of DB, but excessive titanium will lead to the deformities of DB and reduce the mechanical strength of DB.	Ti‐BaldH has the highest mineralization, which can reach more than 5 wt%.	Amorphous oxide nanoparticles are thermally annealed into anatase crystalline titanium oxide.	Non‐uniform distribution on DB, not embedded in DB, mainly deposited on the pore surface of DB.	It can be used for environmental remediation, decomposition of toxic chemicals, preparation of solar cells and so on.	*Pinnularia sp* ^[^ ^97^, ^99^, ^100^ ^]^ *Fistulifera solaris* ^[^ ^101^ ^]^ *Schroederi* *Caloneis* ^[^ ^102^ ^]^
Ca	CaCl_2_	The shape and nanostructure of DB are not affected, and the pores of DB are not blocked.	0.9±0.05 Atomic %	Calcium oxide mostly, not covalently bound to DB.	Calcium is not embedded in DB, but deposited on the surface of the DB.	Hemostatic materials and bone tissue medical materials	*Thalassiosira* *Coscinodiscus sp*.^[^ ^51^ ^]^ *Weissflogii* ^[^ ^106^ ^]^
Zn	Zn‐EDTA	Excessive zinc element makes the DB lose symmetry, resulting in valves and gbs deformity.	The zinc content in DB increased with the increase of zinc content in the medium, and there was a sinusoidal‐dependent relationship between the two.	∖	∖	∖	*Thalassiosira pseudonana* ^[^ ^107^ ^]^ *Stephanodiscus hantzschii* ^[^ ^108^ ^]^
Fe	Fe‐EDTA, Fe‐EDDHA	Without affecting the normal growth of diatoms, there is no significant difference in pore structure and morphology.	The iron content in DB did not increase linearly with the increase of iron content in the medium, and only 1–2% of iron provided in the medium was incorporated into DB.	Most of them are Fe_2_O_3_ clusters and a small amount are Fe^3+^	Dispersed in DB.	Possibly as a catalytic active ingredient.	*Thalassiosira pseudonana* ^[^ ^107^ ^]^ *Stephanopyxis turris* ^[^ ^109^ ^]^
Al	The dirichelate of aluminum	There was no significant morphological change in DB.	Al∖Si is about 1∖15	Aluminum is present in DB as a four‐ or six‐fold coordination amorphous aluminosilicate phase.	It's incorporated into DB	∖	*Stephanopyxis turris* ^[^ ^111^ ^]^
Au	HAuCl_4_	There was no significant morphological change in DB.	∖	Gold nanoparticles	Distributed on the surface of DB.	It can be used as a catalyst.	*Navicula atomus* and *Diadesmis gallica* ^[^ ^105^ ^]^
Eu	Eu(NO_3_)_3_·6H_2_O	There was no significant morphological change in DB.	∖	Eu_2_O_3_ is formed at 600°C and Eu_2_SiO_5_ at 1000°C	∖	Silicate‐based red phosphors can be used in LEDs.	*Navicula* ^[^ ^113^ ^]^

#### 5.2.1 Germanium

Germanium, a homologous element adjacent to silicon, has an atomic structure very close to that of silicon. This makes germanium a strong competing substrate for silicon in the synthesis of DB biosilica, and the presence of germanium can inhibit silicon uptake. The ways in which elemental germanium uses the process of DB biofabrication to alter its structural state are 1) Through the surge uptake mechanism, which induces superior germanium uptake over silicon uptake and keeps the overall shape of DB intact but alters the pore size and geometry of the nanoscale pores of DB.^[^ ^92^, ^93^ ^]^ 2) Mineralization of DB with elemental germanium using a two‐stage incubation method, which usually takes soluble germanium compounds as precursors, eventually forming oxides of germanium on DB (Figure 8B).^[^ ^94^, ^95^ ^]^ Germanium oxides have strong blue photoluminescence, while DB has blue‐green photoluminescence. Thus germanium doping into DB can significantly improve the PL properties of DB.^[^ ^94^ ^]^ The photoluminescence of germanium oxides can be enhanced by converting amorphous GeO_2_ to GeO through thermal annealing treatment.^[^ ^95^, ^96^ ^]^ On the other hand, germanium is also an excellent semiconductor material. The excellent photoelectric and piezoelectric properties of germanium give germanium mineralized DB biosilica materials good potential for semiconductor conduit and microelectronic device applications.^[^ ^94^ ^]^

#### 5.2.2 Titanium

**Figure 9:**
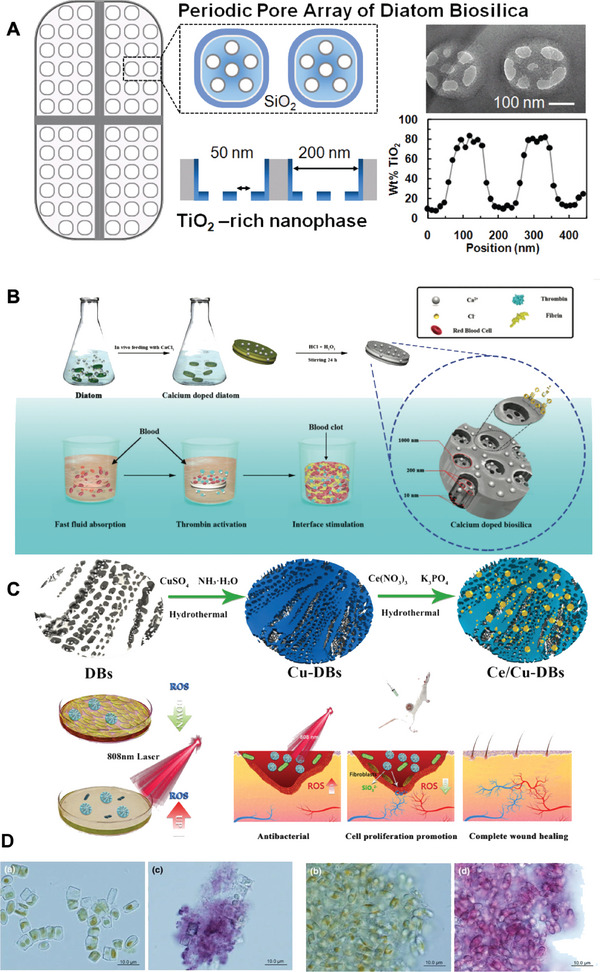
A) Conceptual illustration of TiO_2_‐rich nanophase in frustule biosilica (not to scale). B) Schematic illustrations of the preparation and hemostatic mechanism of calcium‐doped biosilica (Ca‐biosilica). C) Synthesis of Ce/Cu‐DBs and its application. D) Optical microscope images of: Diadesmis gallica a) before and c) after Au (III) addition, and Navicula atomus b) before and d) after Au(III) addition. E) Mechanism for Rh B photodecomposition using CN‐TSD‐2 based on DB. (A) Reproduced with permission.^[^ ^97^ ^]^ Copyright 2008, American Chemical Society. (B) Reproduced with permission.^[^ ^51^ ^]^ Copyright 2013, Royal Society of Chemistry. (C) Reproduced with permission.^[^ ^104^ ^]^ Copyright 2022, American Chemical Society. (D) Reproduced with permission.^[^ ^105^ ^]^ Copyright 2011, Springer Nature.

Diatom DB uptake traceable amounts of certain elements during biofabrication, such as titanium and calcium. Existing in these diatoms traceably, such elements are more readily mineralized onto DB. In the first mineralization of titanium on DB, Ti‐HCl was used as a precursor solution in a two‐stage incubation method.^[^ ^98^ ^]^ A photobioreactor controlled the addition rate of Si and Ti. Titanium as a nanophase deposited on the bottom of each shell pore to form DB biomaterials. Simultaneous provision of sufficient Si was essential to achieve complete uptake of Ti by diatoms (Figures 8C and 9A).

On the one hand, considering the generally low solubility of Ti precursors in water, it is often necessary to adopt a photobioreactor to control the spiking rate. The mineralization effect of different precursor solutions such as Ti(OH)_4_, TiOSO_4_, or Ti‐HCl has been examined: Ti‐BaldH has a higher solubility in water and shows the ability to be independent of the photobioreactor delivery.^[^ ^99^, ^100^ ^]^ On the other hand, the selection of titanium‐tolerant diatom species can also contribute to the effectiveness of mineralization.^[^ ^101^ ^]^ The effectiveness of seven different diatoms to mineralize elemental titanium by biofabrication has been assessed, among which *Caloneis schroederi* shows higher biomass in a two‐stage culture system and has an appropriate exoskeletal rigidity to better mineralize titanium dioxide by biofabrication on DB on mineralized titanium dioxide.^[^ ^102^ ^]^ In addition, genetic engineering means can be taken to increase the amount of mineralized titanium on DB (see Section 4.3 for details), which can increase the amount of titanium mineralized into DB.^[^ ^101^ ^]^ However, excessive amounts of titanium can cause deformities and reduced mechanical properties in DB, affecting the cellular growth of diatoms.^[^ ^99^ ^]^

DB has promising potential for applications in environmental remediation (**Figure** **9**B), decomposition of toxic chemicals, and preparation of solar batteries for energy conversion and storage after being mineralized to titanium and thermal annealing.^[^ ^101^ ^]^ Currently, the main application avenues are 1) Using the photocatalytic properties of titanium‐mineralized DB to treat the environmental pollutant Congo red and chloroform.^[^ ^102^ ^]^ 2) Preparation of co‐doped titanium dioxide DB biomaterials to achieve the decomposition of Rhodamine B in water and disinfection of *Escherichia coli* (Figure 9E).^[^ ^103^ ^]^

#### 5.2.3 Calcium

Calcium is also an element in trace amounts on DB readily to be mineralized.^[^ ^51^, ^106^ ^]^ Calcium is mainly deposited on the surface of DB for mineralization, rather than being covalently bound or embedded in DB. Calcium‐mineralized DB does not change the shape or nanostructure of DB, nor do they block the pores of DB. Moreover, calcium remains firmly in DB after mineralization and acid treatment.

Due to the role of calcium in promoting bone cell growth and acting as a coagulation factor, calcium‐mineralized DB can potentially be used as a hemostat material and a medical material for bone tissue. The existing applications are 1) The calcium‐mineralized DB, prepared by adding CaCl_2_ to a diatom medium, can further promote the growth of human osteogenic sarcoma cells Saos‐2, due to the high affinity of DB for Saos‐2 cells.^[^ ^106^ ^]^ 2) Due to the high specific surface area, porosity, and strong water absorption, DB has inherent hemostat properties. And the combination of DB with calcium, which is a coagulation factor, can exert faster hemostat effects because the calcium doping attenuates the polarity of the silanol groups on the diatom surface, alleviating the higher hemolysis and cytotoxicity of DB.^[^ ^51^ ^]^

#### 5.2.4 Iron and zinc

Both Fe and Zn are trace elements required by diatoms during growth and are incorporated into DB as they divide, proliferate, and metabolize to synthesize DB.^[^ ^107^, ^108^ ^]^ The Zn content of DB increases with the increase of Zn content in the medium and shows a sinusoidal dependence between the two, and the Zn content in DB is also positively correlated with the Zn content in diatom cells, suggesting that the Zn in DB comes from diatom cells.^[^ ^108^ ^]^ As the same zinc mineralization results occur in the diatoms *Stephanodiscus hantzschii* and *Thalassiosira pseudonana*, it can be assumed that zinc is incorporated into the DB of these two diatoms by the same mechanism.

Whereas Fe in DB does not increase with increasing Fe content in the medium, the amount of Fe in DB is strictly regulated by the diatoms themselves, and the amount of Fe deposited in raw DB does not increase linearly with increasing Fe content in the medium.^[^ ^107^, ^109^ ^]^ The growth and viability of diatom cells are not affected when Fe:Si mass ratio is less than 0.5:1, and only 1–2% of the iron provided by the medium is incorporated into the DB biosilica. 95% of the incorporated iron is present in clusters, the majority of which are oxide Fe_2_O_3_ clusters, leaving only 5% in the form of dispersed Fe^3+^.^[^ ^109^, ^110^ ^]^ Hydrated iron oxide modified diatomaceous earth can increase the surface area and improve the adsorption capacity for phosphate. The higher the iron content, the stronger the adsorption capacity. This adsorption is highly selective, and the phosphate adsorption effect is dependent on the pH and ionic strength in the solution. In addition, iron‐modified DB that has already adsorbed phosphate can be reused after alkaline treatment.

#### 5.2.5 Other Metallic Elements

Other metallic elements can also be obtained through biofabrication with corresponding mineralized DB. Mineralization pathways involving Al, Cu, Au, Ag, etc. include: 1) The highest Al mineralization in DB can be achieved when the precursor is a di‐tris chelate of Al, achieving Al: Si of about 1:15, and Al mineralization exists as a quadruple or sixfold coordinated amorphous aluminosilicate phase.^[^ ^111^ ^]^ 2) Gold mineralization with HAuCl_4_ as a precursor results in the synthesis of gold nanoparticles at different positions in the diatoms, regardless of diatom species (Figure 9D).^[^ ^105^ ^]^ 3) Both copper and sulfur‐modified DB materials can significantly improve the light absorption efficiency and the separation and transport efficiency of photogenerated carriers of DB silica materials, with excellent photocatalytic degradation efficiency for methyl blue (MB) and methyl orange (MO).^[^ ^112^ ^]^ Ce/Cu‐DBs can relieve cellular oxidative stress by clearing local excess ROS, while inhibiting bacterial growth by increasing ROS levels under NIR radiation (Figure 9C).^[^ ^104^ ^]^ 4) Europium‐mineralized DB can be used as silicate‐based red phosphors with patterned nanostructures. When applied in LEDs, they have the advantages of chemical stability, moisture resistance and low material cost, which can stimulate the development in the range of phosphors that can be effectively excited by near‐ultraviolet light, particularly those that emit red light.^[^ ^113^ ^]^ 5) Phosphorescent silica clusters less than 100 nm in diameter, obtained by metallic iridium mineralization, have the potential for applications in imaging, sensing, and biomedical photonics.^[^ ^88^ ^]^

### 5.3 Genetic Modification

#### 5.3.1 Genetic Engineering

**Figure 10:**
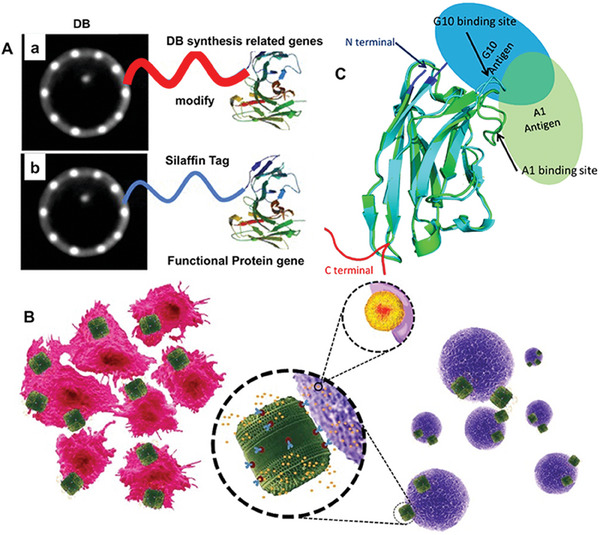
A) Schematic diagram of a) the structure of DB modified by genetic engineering. b) The functional proteins are genetically expressed on the DB. B) The principle of action of the genetically engineered DB therapeutic nanoparticles. Genetically engineered DB (green) containing liposome‐encapsulated drug molecules (yellow) can be targeted to both adherent neuroblastoma cells (red) and lymphocyte cells in suspension (purple) by functionalizing the DB surface with cell‐specific antibodies. C) Aligned homology models of single domain antibodies with presumed antigen binding configuration. The ribbon diagrams of the homology structures of sdAbEA1/A1 (cyan, based on pdb ID 6GLW) and sdAbEA1/G10 (green, based on pdb ID 5F10) were aligned using Pymol. The ovals show the presumed binding positions of the EA1 antigen for sdAbEA1/A1 (light blue) or sdAbEA1/G10 (light green). The N‐ and C‐terminal regions of the sdAbs are also noted in blue and red, respectively. (A) Reproduced with permission.^[^ ^118^ ^]^ Copyright 2016, American Chemical Society. (B) Reproduced with permission.^[^ ^119^ ^]^ Copyright 2015, Springer Nature. (C) Reproduced with permission.^[^ ^120^ ^]^ Copyright 2020, MDPI.

The most basic method to screen the structural parameters of DB is species selection. The morphology and structure of DB can also be changed through chemical modification and biological culture. In addition, gene modification is another method to obtain the required structure of DB.^[^ ^59^ ^]^ To adopt the gene modification method, it is necessary first to identify the genes that guide the formation of DB structure and then modify the genes related to the synthesis of DB (Figure 10A). *Thalassiosira pseudonana*, a model organism in diatom genetic engineering that can self‐assemble transgenic DB in vivo. The genome of this diatom has been deciphered, and the genes that can encode proteins for silicate have sequences that are conserved in several different central diatoms.^[^ ^114^ ^]^ Silacidin protein is one such protein involved in DB cell wall synthesis. Dysregulation of silacidin expression can lead to changes in DB diameter.^[^ ^114^, ^115^ ^]^

Researchers investigated post‐transcriptional regulation during silicification in diatoms, specifically in *T. pseudonana*. They examined untranslated regions (UTRs) and poly(A)‐tags of genes related to DB synthesis using PAT‐seq technology. Their findings showed that alternative polyadenylation (APA) played a significant role in gene expression during various stages of silicon utilization and DB formation. Silica starvation of diatom cells revealed notable APA changes in genes associated with gbs and valves synthesis, indicating their role in silica utilization.^[^ ^116^ ^]^

In addition, to identify effective genes for DB synthesis, researchers developed an image analysis and machine‐learning‐based method. Artificial neural networks (NNs) were used to distinguish wild‐type and knockout mutant diatoms based on scanning electron microscope (SEM) images of DB. This approach, utilizing pore‐related parameters, demonstrated a 94% accuracy in distinguishing between wild‐type and knockout mutant DB, despite the morphological changes resulting from gene knockout.^[^ ^117^ ^]^

#### 5.3.2 Expression of Functional Proteins

Functional proteins can be expressed on DB through genetic modification (Figure 10A), and the resulting functionalized silica materials can be used for drug delivery, sensing, and catalysis. Among the proteins associated with the synthesis of DB, the silaffins protein can enter the silica deposition vesicles SDV to participate in the condensation of silica and become part of the nascent shell layer, so the relevant genes encoding silaffins are usually selected to insert fusion protein genes to express functional proteins on DB. Similarly, *T. pseudonana* is the most commonly used model species in this context.

Researchers have harnessed DB for various applications, including drug delivery, catalysis, and sensing. For drug delivery, they fused signal peptide gene fragments from the silaffin‐3 protein gene with green fluorescent protein (GFP) and antibody binding protein GB1 into the diatom *T*. pseudonana genome. This modification enabled specific binding to B‐cell CD20 antibodies, and DB loaded with hydrophobic anticancer drugs could release therapeutic doses, inducing cancer cell death with site‐specific accumulation.^[^ ^119^ ^]^

In terms of catalysis and sensors, the expression of related proteins, including antibodies and fluorescent proteins, was achieved by inserting genes into DB synthesis‐related genes (Figure 10B). This approach involved using the diatom‐specific expression system and resulted in DB biosilica functionalized with antibodies against Bacillus anthracis and TNT explosives.^[^ ^101^ ^]^ Additionally, green fluorescent protein (EGFP) expressed in DB could differentiate between DB at different formation stages and assess the localization of recombination proteins.^[^ ^118^ ^]^

One optimization involved repositioning the silica‐targeting peptide Sil3T8 to the C‐terminus of single structural domain antibodies (sdAb) to improve antigen access (Figure 10C), enhancing the DB's potential as an environmental biosensor.^[^ ^120^ ^]^ Furthermore, using single‐chain antibodies (scFv) in DB maintained high‐affinity binding in the presence of pro‐solvents, without requiring organic solvents or covalent cross‐linking. By expressing antibody lgG's binding domain on *T. pseudonana*’s surface, hydrophobic anti‐cancer drugs could be encapsulated and targetedly delivered to tumor sites, effectively killing tumor cells while sparing healthy tissues. The high protein content in DB contributed to the stability of functional proteins in challenging environments.^[^ ^121^ ^]^

These innovative applications of DB have the potential to transform environmental sensing biomaterials and provide promising solutions for drug delivery and targeted therapies in cancer treatment.

## 6 Conclusion and Outlook

Current studies can select DB from different algal sources for comparative analysis, but the needle‐in‐a‐haystack model of searching from hundreds of thousands of diatom species faces constraint points even if it is completed: the improvement of biomass and the limitation of large density culture. And the translation of this needle‐in‐a‐ seaway selection model into a biofabrication process based on diatom DB themselves, which can promote the scale production and industrial transformation of key raw materials for DB biomaterials. Starting from the inherent micro‐nanolevel porous structure of diatoms and the biofabrication process, the mutual possibilities between DB biofabrication and biomaterial applications are investigated from several perspectives, which are crucial for the further development of DB applications. The biofabrication process plays a decisive role in the subsequent application and multidirectional and multifunctional exploitation of DB biomaterials. It has been demonstrated that the micro‐nanolevel porous structures of DB can be modified directly or indirectly through biofabrication and play an essential role in drug delivery, hemostasis, and bone tissue repair applications.

The multilayered micro‐nanostructures of DB, which can be modulated by biofabrication processes through alteration of external culture conditions and surface modifications of organics or metals, hold promising applications in the biomedical field. Notably, modulation control of the micro‐nanolevel porous structure and elemental composition of diatom DB is critical for changing the direction of DB structure‐dominated applications. Specifically, the basic structural characteristics of DB biomaterials, including pores, size, shape, and surface modifications, should be tuned to conform to the required optimization. In addition, understanding the biofabrication process of diatom DB structures is also essential for tuning DB structures and expanding DB biomaterials applications. The biofabrication process of diatom DB follows the cell cycle of diatoms, and it is important to regulate the biofabrication process of DB from several aspects, including external factors such as silicate nutrients, metallic elements, and genetic modifications, which are important for the regulation and selective functionalization of DB structures.

Significant achievements have been made in the direction of structurally mediated biomaterial applications of DB. In experiments, by adding appropriate precursors such as metallic elements and organic molecules to diatom media, corresponding mineralized DB are obtained, which retain the original structural composition of DB but also possess unique properties associated with metallic elements or organic molecules. At present, titanium‐mineralized DB as solar cells and photocatalytic environmental remediation materials through the mineralization of the biofabrication process; calcium mineralized DB as bone tissue repair materials and hemostat materials; and diatom biosilica optical materials doped with rhodamine dyes, PDMPO, PE dyes, etc. have been obtained. In addition, inserting fusion protein genes into DB synthesis‐related protein silaffins is currently the most common method of expressing related functional proteins on DB. The antibody proteins and fluorescent proteins expressed on the surface of DB enable DB to be used in drug delivery and controlled release, biosensing platforms, industrial catalysis, etc. The means to modify the structure of DB by modifying genes has yet to be developed and studied. Therefore, further research on modifying DB structure by gene modification means can break the shackles of structural regulation and functional innovation of DB as biomaterials. The analysis of the biofabrication and structural composition of DB by genetic means is conducive to penetrating the understanding of the structural and functional plasticity of DB and the rational design of DB biomaterials, which has a positive effect on promoting the structural and functional innovation of DB and provides potential strategies for designing forward‐looking DB biomaterials or even expanding the application directions.

Multiple demonstrations of the feasibility of structural modification of DB through biofabrication processes can be used to develop potential DB biomaterials for further research and development, and different methods can be combined. The genome of the diatom *Thalassiosira pseudonana*, currently commonly used as a model organism in diatom genetic engineering, has been deciphered, and this will be a fateful step in the future to further modulate the structure and function of DB biomaterials systematically and to improve the safety and operability of DB biomaterials in clinical practice. In addition, exploring the combination with other technologies using the biofabrication process of diatoms will also maximize the research value of DB as biomaterials.

## Conflict of Interest

The authors declare no conflict of interest.
